# Harvesting Environmental Microalgal Blooms for Remediation and Resource Recovery: A Laboratory Scale Investigation with Economic and Microbial Community Impact Assessment

**DOI:** 10.3390/biology7010004

**Published:** 2017-12-29

**Authors:** Jagroop Pandhal, Wai L. Choon, Rahul V. Kapoore, David A. Russo, James Hanotu, I. A. Grant Wilson, Pratik Desai, Malcolm Bailey, William J. Zimmerman, Andrew S. Ferguson

**Affiliations:** 1 Department of Chemical and Biological Engineering, The University of Sheffield, Sheffield S1 3JD, UK; liong_waichoon91@hotmail.com (W.L.C.); r.kapoore@sheffield.ac.uk (R.V.K.); j.hanotu@sheffield.ac.uk (J.H.); grant.wilson@sheffield.ac.uk (I.A.G.W.); p.desai@sheffield.ac.uk (P.D.); w.zimmerman@sheffield.ac.uk (W.J.Z.); andrew.ferguson@sheffield.ac.uk (A.S.F.); 2 Copenhagen Plant Science Centre, Department of Plant and Environmental Sciences, University of Copenhagen, Thorvaldsensvej 40, C 1871 Frederiksberg, Denmark; russo@plen.ku.dk; 3 Link2Energy, 1-3 Bigby Street, Brigg, North Lincolnshire DN20 8EJ, UK; malcolm@link2energy.co.uk

**Keywords:** environmental microalgae, resource recovery, eutrophication, microflotation, algal bloom

## Abstract

A laboratory based microflotation rig termed efficient FLOtation of Algae Technology (eFLOAT) was used to optimise parameters for harvesting microalgal biomass from eutrophic water systems. This was performed for the dual objectives of remediation (nutrient removal) and resource recovery. Preliminary experiments demonstrated that chitosan was more efficient than alum for flocculation of biomass and the presence of bacteria could play a positive role and reduce flocculant application rates under the natural conditions tested. Maximum biomass removal from a hyper-eutrophic water retention pond sample was achieved with 5 mg·L^−1^ chitosan (90% Chlorophyll *a* removal). Harvesting at maximum rates showed that after 10 days, the bacterial diversity is significantly increased with reduced cyanobacteria, indicating improved ecosystem functioning. The resource potential within the biomass was characterized by 9.02 μg phosphate, 0.36 mg protein, and 103.7 μg lipid per mg of biomass. Fatty acid methyl ester composition was comparable to pure cultures of microalgae, dominated by C16 and C18 chain lengths with saturated, monounsaturated, and polyunsaturated fatty acids. Finally, the laboratory data was translated into a full-size and modular eFLOAT system, with estimated costs as a novel eco-technology for efficient algal bloom harvesting.

## 1. Introduction

The world’s growing population is putting pressure on natural resources and increasing waste production. Current rates and practices will deplete finite resources whilst potentially causing irreversible pollution to natural ecosystems. Nutrient release (e.g., nitrogen (N) and phosphorus (P)) is a major source of anthropogenic pollution. Sources of these nutrients include fertilizers, sewage and industrial effluents [1]. Most of these nutrients accumulate within aquatic ecosystems, leading to eutrophication of static water bodies including lakes and reservoirs. Indeed, 30–40% of global lakes and reservoirs are now classified as eutrophic, leading to algal blooms [2] that can be toxic, reduce biodiversity, damage ecosystem health, and kill wildlife. Moreover, climate change is expected to heighten the problem [3]. “Mega-algal blooms” have been recorded in the USA, where the economic cost has been estimated at $2.2 billion per year [4,5], and a single bloom event in Lake Taihu was estimated to have cost the Chinese economy $6.5 billion [6].

Efforts to remediate eutrophic water systems are varied and limited in success. Reducing external nutrient loading is the preferred option. This system relies on identifying a point source, or if the problem is diffuse pollution (e.g., from agriculture or urban areas), the task becomes more complex. Options to reduce diffuse pollution include catchment scale management driven by policy [7]. As well as involving local stakeholders, a major concept is ‘slow water’, i.e., slowing flow of water from land into watercourses through the use of constructed wetlands or ‘buffer strips’. Even if reduced external loading is achieved, P can be released from sediments (legacy P) for many years [8]. Biomanipulation is difficult to control and predict due to the instability of aquatic communities and the fundamental problem remains that the nutrient is not removed. In some countries, notably the USA, nutrients are locked in the sediment using chemicals, but similar issues with long-term P release from sediment occur [9]. There have also been recent efforts to install filamentous algal-based scrubbing systems to remove nutrients from agricultural storm water [10]. An alternative option is to harvest the environmental algal biomass, thus removing a proportion of the nutrients that reside within algal cells to help break the continual eutrophication loop caused by internal nutrient loading or diffuse pollution. Although algal harvesting should be considered as an addition to the lake restoration toolbox, the associated energy costs can be prohibitive. As experienced by the algal biodiesel community, up to 30% of the overall process costs can be attributed to harvesting microalgal cells [11]. Accordingly, sedimentation and flotation are generally considered the most viable low-cost harvesting methods for large volumes of microalgal biomass. In regards to flotation, dissolved air flotation (DAF) is a well-established process for separating suspended particles from the liquid phase, by bringing the particles to the surface [12]. Microbubbles are formed when water, saturated with air at high pressure, is released from a diffuser. These then attach to flocculated particles rising to the surface [12]. This process is applied across many industries e.g., domestic wastewater treatment, oil refineries, and metal ore processing. However, these industries work within closed systems, where conditions can be manipulated to optimize harvesting efficiencies. Moreover, due to DAF requiring high-pressure nozzles, or more recently methods such as ultrasound [13], energy costs are relatively high, making the application to large amounts of water, such as a eutrophic lake or retention pond, prohibitive. Indeed, wastewater aeration using microbubbles has been estimated to consume up to 0.4% of the UK’s total energy consumption [14].

Recently, a device termed a fluidic oscillator (FO) that exploits microfluidic principles by converting laminar air flow into oscillatory flow, followed by ejection of microbubbles through a diffuser, has shown considerable promise for widening the applications of microbubbles [15]. Bubble production has been demonstrated at a large-scale (2200 L) in a continuous state operation (tested to 17 days) [16]. With no requirement for pressurization and liquid pumping, the process only uses a fraction of the energy of traditional DAF methods [17]. Microflotation using the FO has been applied to harvest pure cultures of yeast and dead microalgal cells [16,18] but has not been applied to environmental samples with live microalgae and bacterial consortia. Therefore, we incorporated the FO into the design of a laboratory-scale environmental algae harvesting device, termed efficient FLOtation of Algae Technology (eFLOAT) (Supplementary Figure S1).

This study seeks to examine the potential of eFLOAT to harvest environmental biomass from a bloom event, conducted under the constraint of near-natural environmental conditions. Through a series of laboratory-based experiments, data were generated for a proof of concept for the flocculation and subsequent removal of algal blooms (and nutrients) from lakes. Preliminary sedimentation trials aimed to compare flocculation efficiencies to (1) compare an environmentally sourced lake sample with pure microalgae cultures to assess for differences in flocculant doses; (2) compare a common chemical flocculant (aluminium sulphate or alum) with a biopolymer flocculant (chitosan), where the former has been associated with risks to aquatic life and is known to impact on the downstream use of recovered algal biomass; and (3) gain insight into the effects of having more bacteria present. Subsequently, a sedimentation test was undertaken using P-rich water sourced from a water retention pond (WRP), owned by a UK water company and known to experience regular algal blooms. This was performed to select two conditions for maximum and sub-maximum harvesting of microalgae using eFLOAT. The eFLOAT experiment was undertaken and P-removal rates were calculated. In terms of an environmental remediation technique, Chlorophyll *a* (Chl *a*) levels were compared pre- and post-harvesting after a prolonged period (10 days), using a non-harvested sample as a control. The bacterial and eukaryotic diversity was also characterized within these samples, as an increase in microbial diversity was considered an indication of improved ecosystem function and, therefore, health [19].

Although environmental remediation is the main focus of eFLOAT, the advantages can be further improved if the biomass can be used as a resource. Recently, microalgae have attracted significant interest globally as a potential feedstock for the bio-based economy [20]. Here, we characterize and quantify three biochemical constituents (lipids, phosphates and proteins) in the recovered biomass, which represent different potential resource streams including precursors for industrial chemicals, fuels, fertilizers, and protein feed. The focus here is microalgae and not potentially toxic cyanobacterial blooms. Finally, it is of interest to consider the requirements of a full size eFLOAT modular device. Therefore, an operating and capital expenditure was undertaken based on the findings of the laboratory experiments and data within the literature, incorporating a solar-powered eFLOAT system.

## 2. Materials and Methods

### 2.1. Sample Collection 

Environmental samples were collected using a white, opaque, polypropylene vessel. For initial sedimentation tests, a 2 L water sample was collected from Weston Park Lake (WPL), Sheffield, UK (53°22′56.849′′ N, 1°29′21.235′′ W), on 16th April 2014 during a bloom event. For subsequent sedimentation, eFLOAT, and resource analysis, 50 L of water was collected from a water retention pond (WRP) in the UK during a bloom event on 19th May 2014. 

### 2.2. Measurement of Water Retention Pond Abiotic and Biotic Variables

The temperature, pH, dissolved oxygen (DO), conductivity and salinity of WRP sample were measured with a Professional Plus Quatro (YSI, Yellow Springs, OH, USA). A 50 mL aliquot was collected, filtered with a Minisart High Flow 0.45 µm syringe filter (Sartorius, Germany), and analysed for ammonium (NH_4_^+^), bromide (Br^−^), calcium (Ca^2+^), chloride (Cl^−^), fluoride (F^−^), magnesium (Mg^2+^), nitrate (NO_3_^−^), nitrite (NO_2_^−^), phosphate (PO_4_^3−^), potassium (K^+^), sodium (Na^+^) and sulphate (SO_4_^2−^) concentrations using a Dionex ICS-3000 ion chromatograph (Thermo Fisher Scientific, Waltham, MA, USA). Anions were measured on an AG18 2 × 250 mm column with a flow rate of 0.25 mL·min^−1^ and 31.04 mM potassium hydroxide as eluent. Cations were measured on a CS16 4 × 250 mm column with a flow rate of 0.36 mL·min^−1^ and 48 mM methanesulfonic acid as eluent. To estimate microalgae (and cyanobacterial) abundance, Chlorophyl a (Chl *a*) concentrations were calculated using methanol and glass bead cell disruption as outlined by Welburn et al. [21]. For dry cell weight measurement, a 50 mL aliquot of WRP was centrifuged at 3000× *g* for 10 min at 4 °C, frozen at −20 °C and immediately freeze-dried in a Modulgo freeze dryer (Edwards, Crawley, UK) for 12 h and weighed.

### 2.3. Pure Microalgae Cultivation and Preliminary Sedimentation Tests

*Chlamydomonas reinhardtii* (CC-125, Chlamydomonas Resource Centre, University of Minnesota, Minneapolis, MN, USA), and *Chlorella vulgaris* (CCAP 211/12, Culture Collection of Algae and Protozoa, Oban, Scotland) strains were chosen for the pure culture tests because they are both laboratory representative strains of green algae. Cells were cultured using artificial freshwater growth medium (Table S1) in 250 mL conical flasks and under 70 µmol m^−2^·s^−1^ illumination (12 h light, 12 h dark) on an orbital shaker at 110 rpm. Diluted to the same optical density (600_nm_ = 0.5), both algal cultures and the WPL sample were subjected to two coagulating agents, alum, Al_2_(SO_4_)_3_ (12.5 mg·L^−1^ to 100 mg·L^−1^) and chitosan (1 mg·L^−1^ to 6 mg·L^−1^) [18] by rapid mixing in a jar tester (Flocculator S6, Stuart, FL, USA) at 250 rpm for 10 min, followed by slow mixing at 100 rpm for 5 min, to promote cell aggregation. To create an environmental sample with a reduced bacterial concentration, 1 L of WPL water was centrifuged at 3000× *g* for 15 min to produce biomass-free water. A separate 1 L of WPL water was filtered with a 3 µm pore size polycarbonate isopore membrane (EMD Millipore, Burlington, MA, USA) which would recover mostly algal biomass while allowing smaller free-living bacteria to pass through [22]. The recovered algae were subsequently re-suspended in 1 L of the biomass-free water. Bacterial cell numbers were verified using a Bright-Line glass haemocytometer (Hausser Scientific, Horsham, PA, USA) on a BX 51 microscope (Olympus, Tokyo, Japan). A paired *t*-test was applied to identify statistically significant changes.

### 2.4. WRP Sample Sedimentation and eFLOAT

The WRP sample was flocculated with chitosan using the aforementioned procedure with concentrations of 1 to 9 mg·L^−1^. This range of concentrations was tested to select two sedimentation efficiencies for the microflotation stage, a high sedimentation efficiency (SE_max_) and a lower efficiency (SE_sub-max_). Following flocculation, eFLOAT was applied temporally at both SE_max_ and SE_sub-max_ chitosan concentrations (including a blank where no chitosan was added), sampling every 2 min for 10 min (in duplicate). A laboratory-scale eFLOAT system was constructed from a Perspex flotation column, measuring 9 cm in diameter × 30 cm in height, with a microbubble ceramic diffuser (Point 4 MBD systems, mesoporous ceramic, 2–10 µm pore size, with SEM picture provided by Brittle et al. [23]) and sampling port (Supplementary Figure S1). A 2.2 kW compressor with cylinder size 0.1 m^3^ and duty cycle (50%) 4 bars was used to pass compressed air through the fluidic oscillator and a portion of the air (1 mL·min^−1^) fed into the microporous diffuser, where it exited as microbubbles. During eFLOAT, 1 L of the sample was poured into the flotation column before introducing microbubbles. 

### 2.5. Biochemical Composition

Analyses of the biochemical composition of the WRP sample was undertaken using methods described previously [24]. Briefly, a predetermined volume of the environmental samples were filtered onto a precombusted 13 mm A/E glass fiber filter paper (Pall Corporation, Cortland, NY, USA) at room temperature, dried at 60 °C for 24 h, wrapped in tin discs (Exeter Analytical, UK) and analyzed for total nitrogen (N) and total carbon (C) using a SerCon GSL elemental analyser (1000 °C) interfaced with a 20–20 Isotope Ratio Mass Spectrometer (PDZ–Europa, Northwich, Cheshire, UK). Isoleucine standards with known C:N ratios were used to calibrate measurements. Protein content was calculated by a nitrogen to protein conversion (4.78) [25]. Results were averages of three technical replicates. Soluble organic P measurements were made by converting organic P from the environmental biomass on precombusted glass filters to orthophosphate, by digesting with acidic persulfate. A phospho-molybdate assay was used for measurements at 880 nm [26]. Total lipids were measured gravimetrically using a method based on chloroform and methanol [27] with modifications [24].

### 2.6. Fatty Acid Methyl Ester Composition

All chemicals and analytical reagents were of high performance liquid chromatography grade (Sigma–Aldrich, Dorset, UK) unless stated otherwise. Biomass samples (15 mL) were pelleted by centrifugation at 19,000× *g* for 3 min to which 1.2 mL of a methanol:chloroform (1:2, *v/v*) and equal volume of glass beads (425–600 µm, acid washed) were added. Cells were disrupted with a Genie cell disruptor (Scientific Industries Inc., Bohemia, NY, USA) for 15 cycles (1 min bead beating and 1 min stand in ice bath). After cell disruption, the supernatant was collected after centrifugation at 19,000× *g*, at 4 °C for 10 min and added to 800 μL of chloroform and water (1:1 *v/v*). After further centrifugation (8000× *g* at 4 °C for 10 min) the organic phase was pre-weighed prior to evaporation under inert nitrogen gas using a six port mini-vap evaporator (Sigma-Aldrich, Dorset, UK) and stored at −80 °C until further analysis. The extracted lipids were converted into fatty acid methyl esters (FAMEs) [28] with minor modifications: 250 μL of chloroform:methanol (1:1, *v/v*) and 100 μL of 10% (*w/v*) BF_3_/methanol was added to the dried extract and incubated at 80 °C for 90 min. After cooling, 300 μL water and 600 μL hexane were added, centrifuged (18,000× *g* at 4 °C for 10 min) and 500 μL of the organic phase removed and evaporated to dryness under inert nitrogen gas. The dried FAMEs were reconstituted in 100 μL hexane prior to identification and quantification on a TRACE 1300 gas chromatography flame ionization detector (GC-FID) System (Thermo Scientific, Hertfordshire, UK) using a TR-FAME capillary column (25 m × 0.32 mm × 0.25 µm). 1 µL derivatized sample was injected in split injection mode at 250 °C (split flow 75 mL·min^−1^ and purge flow 5 mL·min^−1^). The GC-FID was operated at a constant flow of 1.5 mL·min^−1^ helium at an initial temperature of 150 °C for 1 min, followed by ramping at 10 °C·min^−1^ to 250 °C and held constant here for 1 min. Peak identities were ascertained using an external standard 37 component FAME mix (Supelco, Bellefonte, PA, USA) and peak areas were integrated using a chromatography data system (Thermo Scientific Dionex ChromeleonTM 7 software, Version 7.2.0.4154). In total, five technical replicates were run, among which only the FAMEs identified in 3 or more replicates were considered true hits. 

### 2.7. Microbial Diversity Analysis (16S and 18S rDNA Gene Sequencing) 

DNA extractions for microbial diversity analysis were performed on biomass taken from (i) WRP (day 0), (ii) non-chitosan treatment blank (day 10) (iii) Sub-max chitosan treatment (day 10) and (iv) max chitosan treatment (day 10). Conditions (ii) to (iv) were undertaken in duplicate. DNA was extracted with a standard phenol-chloroform extraction protocol (Sambrook and Russel, 2001). PCR amplification, product pooling, purification and sequencing were performed by RTL Genomics (Lubbock, TX, USA) using an Illumina MiSeq (Illumina, Inc. San Diego, CA, USA) and as described previously [29]. Bioinformatic and statistical analysis involved merging the forward and reverse reads [30] and filtering for quality and clustering using the USEARCH algorithm [31]. Chimeras were removed using the UCHIME chimera detection software executed in de novo mode [32] with reads mapped using the USEARCH global alignment algorithm [31]. Searches were performed using an in-house curated database retrieved from NCBI (17th October 2015). Finally, the OTU table output from sequence clustering was collated with the taxonomic information [33] as described previously [29]. The SEED algorithm was applied to create PCA plots for genus level taxa present above 1% relative abundance [34]. Shannon diversity indices (H’) were calculated to express diversity within the samples as described previously [35]. 

## 3. Results

### 3.1. Preliminary Sedimentation Tests with Pure Microalgae and WPL Sample

**Figure 1:**
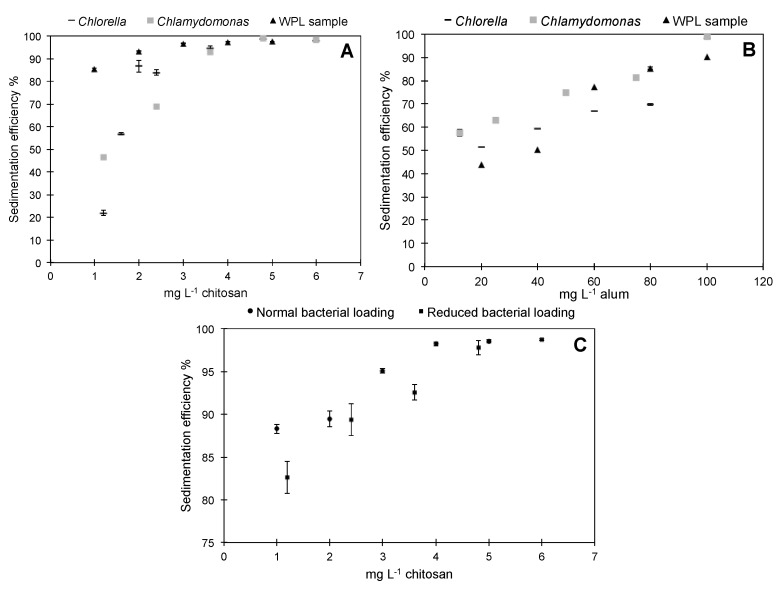
Preliminary sedimentation efficiency experiments using (**A**) Chitosan with *Chlorella*, *Chlamydomonas* and WPL sample, (**B**) Alum with *Chlorella*, *Chlamydomonas* and Weston Park Lake (WPL) sample and (**C**) Chitosan with WPL sample with normal and reduced bacteria concentration (error bars are standard deviation *n* = 3).

Flocculation efficiencies were calculated for the removal of pure algal cultures (*Chlorella* and *Chlamydomonas*) and an environmental biomass sample from WPL (pH 7) using a range of chitosan and alum concentrations (Figure 1). Lower concentrations of chitosan were required to achieve higher flocculation efficiencies with WPL (Figure 1A). The 1 mg·L^−1^ sample of chitosan achieved 85% Chl *a* removal from the WPL compared with just 46% and 22% removal from *Chlamydomonas* and *Chlorella* cell cultures, respectively. Interestingly, the same effect was not observed for alum. When compared to the WPL samples both *Chlorella* and *Chlamydomonas* cells flocculated with significantly higher efficiency at concentrations of alum ranging from 12.5–100 mg·mL^−1^ (*p* < 0.01). Similar to a previous study, these preliminary tests suggest chitosan is more efficient for flocculation of the WPL sample, under the conditions tested [36].

It has been demonstrated previously that bacteria within algal cultures can aid the flocculation process by forming microalgal bacterial flocs inducing sedimentation [37]. In this study, we hypothesized that the presence of bacteria within the WPL sample could improve flocculation efficiency when adding a flocculant, as the resulting bacterial surface charge neutralization could combine with algal cells to form larger aggregates and hence aid sedimentation. In addition, the extracellular metabolites and/or proteins within the water could act as organic polymers to promote flocculation. We compared chitosan-induced flocculation with WPL samples containing natural and reduced concentrations of bacteria. Microscope images showed an 82 ± 13% decrease in bacterial cell numbers using the filtering method. The presence of more bacteria implied a positive effect and significantly improved flocculation efficiency at chitosan doses of 1 mg·mL^−1^ (*p* = 0.027) with no significant difference in concentrations above 2 mg·mL^−1^ (Figure 1C). Further work would need to be undertaken to distinguish between the specific roles of both extracellular metabolites and bacteria in flocculation.

### 3.2. WRP Pond Sedimentation Test and eFLOAT

**Figure 2:**
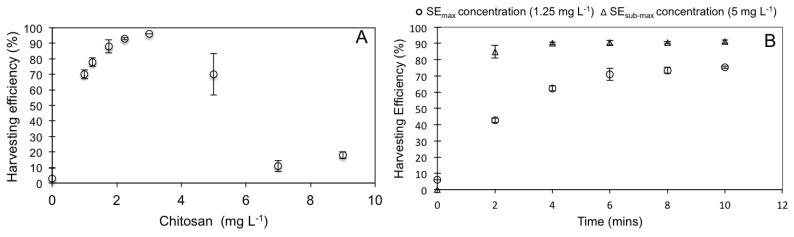
WRP sample tests (**A**) Sedimentation efficiency using chitosan (**B**) eFLOAT harvesting efficiency at SE_max_ and SE_sub-max_ chitosan concentrations.

The final sedimentation test was undertaken to identify the chitosan concentrations that would provide maximum sedimentation efficiency (SE_max_) and a reduced sedimentation efficiency (SE_sub-max_) with the WRP sample. A SE_sub-max_ chitosan concentration was calculated to alter the application methodology of eFLOAT, where reduced sedimentation efficiency can, potentially, lower application costs and still provide resource and environmental recovery. Also, the reduced harvesting would lessen the impact of the non-selective harvesting approach, and potentially allow the ecosystem to recover more rapidly from eFLOAT. Based on these results, chitosan concentrations of 1.25 and 5 mg·L^−1^ were selected for the eFLOAT experiment as they represented a 70% (SE_sub-max_) and 96% (SE_max_) sedimentation efficiency, respectively (Figure 2A).

The physicochemical characteristics of the WRP are shown in Table 1. A combination of temperature (18.1 °C), season (Spring), and extremely high PO_4_^3−^ levels (6.11 mg·L^−1^) contributed to a high concentration of Chl *a* at 443.32 μg·L^−1^. A time-series eFLOAT experiment was undertaken with this sample using SE_max_ (5 mg·L^−1^) and SE_sub-max_ (1.25 mg·L^−1^) chitosan concentrations (Figure 2B). After 10 min, the harvesting efficiencies using microflotation for SE_max_ and SE_sub-max_ concentrations of chitosan were 91.2% and 75.3%, respectively. These harvesting efficiency values were similar to those seen in the sedimentation test (Figure 2A), and therefore provided validation for using sedimentation data to guide eFLOAT parameters for WRP samples. The major difference between SE_max_ and SE_sub-max_ in the eFLOAT experiment was the time taken to reach the highest harvesting efficiency. Within 2 min SE_max_ led to 84.9% harvesting efficiency, whereas SE_sub-max_ levels only achieved a harvesting efficiency of 42.7%. By 10 min both SE_max_ and SE_sub-max_ chitosan concentrations were more similar (harvesting efficiencies of 91.2% and 75.3%, respectively). This implies that, given enough time, using a third of the higher chitosan concentration value can still recover up to 75% of the environmental biomass in the eutrophic water system tested. It is important to note that due to the mechanism of chitosan-induced flocculation (i.e., adsorption and charge neutralization between positively charged chitosan in water, and negatively charged algal/bacterial cell surfaces [38]), these chosen parameters are highly dependent on the nature of the environmental sample in terms of total biomass, species present, growth stage (i.e., variable cell surface characteristics affecting zeta potentials) and water physicochemistry (e.g., pH, temperature, salt ions etc.) [39]. This is why preliminary investigative sedimentation experiments are routinely undertaken by the water treatment industry. The time of year when the bloom occurs is also an important factor, as microalgae tend to dominate early spring and summer blooms, whereas cyanobacteria are more prevalent during late summer.

### 3.3. Resource Analysis

Considering a fluctuating volume of water within the WRP phototrophic zone of 5000 to 10,000 m^3^, and assuming an even distribution of algal biomass during a bloom event, a dry biomass weight of 0.13 g·L^−1^ equates to approximately 650 to 1300 kg biomass dry weight for the WRP. A relatively high Chl *a* concentration of 443.23 μg·L^−1^ was not unexpected as the PO_4_^3−^ concentration was 6.11 mg·L^−1^ (Table 1). This high concentration is likely due to high levels of agricultural nutrient run-off from surrounding areas.

Arguably, the simplest (from a regulatory and technical perspective) use of environmental waste biomass is recycling to agricultural land (soil restorer) or as a feedstock in anaerobic digesters (biomethane production). However, microalgae are a highly diverse group of organisms with an assorted array of uses [20]. Their lipids can be readily converted to biodiesel [40] and also provide precursors for industrial chemicals [41,42,43,44]. Their cells can be processed to extract phosphorus for fertilizer [45,46] and algal protein has been found to be an attractive food replacement for the unsustainable feedstock presently used for fish and farm animals [47], although regulatory issues would need to be considered here. To gain insight into the resource potential, a biochemical analysis of cells from the WRP sample was undertaken (total lipid, P, and proteins). Furthermore, the FAME composition was characterized and compared to values from pure cultures within the literature (Table S2). 

#### 3.3.1. Total Lipids and FAME Analyses

Prior to FAME analysis, total lipid content in the WRP biomass was calculated at 103.7 ± 1.28 μg·mg^−1^ biomass using the gravimetric method. Considering the estimated total biomass of 650 to 1300 kg dry weight within the WRP, and the lipid content within its biomass, maximum harvesting using eFLOAT would potentially recover 61–123 kg lipids and sub-max harvesting 51–102 kg lipids. 

The fatty acid composition of the environmentally-sourced biomass in this study was analyzed and compared to FAME composition analyses of pure cultures that have been published in the literature (Table S2). The general pattern is similar to pure cultures with the chain length being dominated by C16 and C18 fatty acids. This was expected, as microalgae typically produce saturated and unsaturated versions with these chain lengths [41]. There were differences, for example, 19.3% of WRP biomass contained C14:0 FAME, considerably higher than typically seen in the previously published studies with freshwater microalgae, and more typical of marine species such as *Isochrysis galbana* (14.4%), *Emiliana huxleyi* (18.8%) *and Nannochloris* sp. (13.3%) (Table S2). This general overview demonstrates that the FAMEs extracted from environmentally sourced algal biomass have comparable composition to pure algal cultures. Despite this, environmental conditions play a significant role in the FAME composition of microalgal cells [48], and a more comprehensive understanding of this phenomenon could be used to identify the most appropriate resource type for harvested biomass.

**Figure 3:**
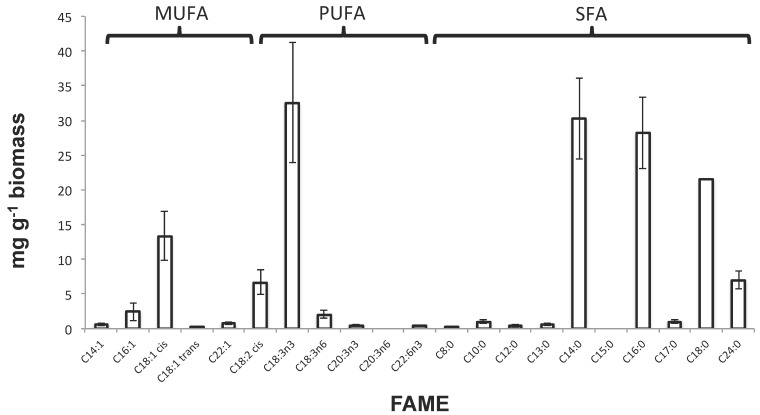
Total FAMEs within biomass sample, characterized as monounsaturated fatty acids (MUFAs), polyunsaturated fatty acids (PUFAs) and saturated fatty acids (SFAs).

To further investigate FAME-related resource potential of the WRP biomass, they were grouped into monounsaturated fatty acids (MUFAs), polyunsaturated fatty acids (PUFAs), and saturated fatty acids (SFAs) by productivity yields (Figure 3), as they represent different potential resources. The biomass had relatively high levels of the MUFA C18:1 cis or oleic acid (13.3 mg·g^−1^ biomass). Oleic acid is commonly found in olive oil and in its sodium salt form it is used as an emulsifying agent within soaps. It has also been used within cosmetic products for its moisturizing qualities [49]. The biomass was also relatively rich in PUFA C18:3n3 (32.5 mg·g^−1^ biomass). In addition, C18:3n3 or α-linolenic acid is an omega-3 fatty acid, essential for the human diet and found in seeds, nuts, and vegetable oils. 

The largest values in terms of mg·g^−1^ biomass were seen for groups of SFAs including C14:0, C16:0 and C18:0 (30.29, 28.17 and 21.5 mg·g^−1^ biomass, respectively). This was perhaps not surprising considering C16:0 or palmitic acid is the most common SFA present in plants, animals, and microorganisms. Industrially, palmitic acid has many uses, for example, in the manufacture of detergents or cosmetics, and is mostly sourced from palm oil. C14:0 or myristic acid is used to synthesize flavor and is an ingredient in soaps and cosmetics, whereas C18:0 or stearic acid is widely used within soaps, cosmetics, and detergents through saponification of TAGs from stearic acid esters.

#### 3.3.2. Phosphorus Content Analyses

The nutrient P accumulated within the biomass was also considered a recoverable resource. The amount of PO_4_^3−^ present in the water sample (Table 1), as well as soluble organic-P present within the recovered algal biomass, was quantified. The WRP contained a concentration of PO_4_^3−^ (6110 µg·L^−1^) and perhaps unsurprisingly its biomass also contained a high concentration of 9.02 µg·mg^−1^ biomass. Considering the estimated total biomass of 650 to 1300 kg dry weight within the WRP, maximum harvesting using eFLOAT could potentially recover 5.35–10.69 kg PO_4_^3−^ and sub-max harvesting 4.41–8.83 kg PO_4_^3−^. The removal efficiency of PO_4_^3−^ in the P-rich WRP sample during the 15 min eFLOAT experiment was calculated to be 14.4% (sub-max) and 17.5% (max), indicating that repeat harvesting could be undertaken as P-levels would be high enough to support further blooms. 

#### 3.3.3. Protein Content Analyses

Algae have been coveted as a protein feed since comprehensive studies on pure cultures have demonstrated that they have high nutritional quality [50]. The protein content of recovered algal biomass was quantified at 325.45 ± 28.63 μg·mg^−1^ biomass. Based on the estimated biomass within the WRP, a maximum harvesting using eFLOAT would potentially recover 192.93–385.69 kg protein at the time of sampling, and sub-max harvesting would recover 159.29–318.50 kg protein. The exact recoverable and usable protein resource would depend upon the specific protein characteristics and efficiency of downstream processes for extraction and purification from algal biomass.

#### 3.3.4. Microbial Diversity Analysis

The importance of the relationship between microbial community diversity and multiple ecosystem functions has only recently been investigated due to advanced molecular sequencing techniques. A recent study provided empirical evidence that any loss in microbial diversity can lead to reduced multi-functionality, negatively impacting any ecosystem services provided [19]. Microbial diversity has been used as a bio-indicator for aquatic ecosystem health previously [51], and algal blooms have been linked to reduced biodiversity. Recently, we have shown that bacteria-driven function is impacted by the nutrient status of aquatic environments [29]. Moreover, despite the motivation of eFLOAT being the removal of polluting algal biomass, the harvesting parameters identified in Section 3.1 and Section 3.2 could target specific microbial groups. To assess the impacts of the sub-maximum and maximum harvesting parameters on microbial composition and diversity, 16S rDNA and 18S rDNA sequencing analysis was undertaken at Day 0 and 10 days after sub-maximum and maximum harvesting had taken place. A control was also included where no harvesting treatment was performed. This data was supported by Chl *a* measurements. As expected, Chl *a* measurements for the blank treated samples were very similar to the initial concentrations in the WRP (395.7 ± 12.2 ug·L^−1^ versus 443.23 ug·L^−1^ respectively). Sub-maximum and maximum harvesting reduced overall Chl *a* by 65.2 ± 2.8% and 82.2 ± 5.4%, respectively (Table S3). Hence, the harvesting did remove a large proportion of the algal biomass, and this remained the case 10 days post-treatment. 

**Figure 4:**
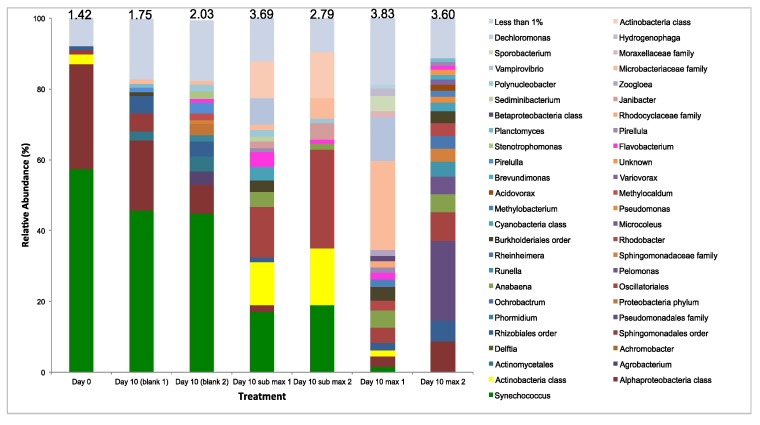

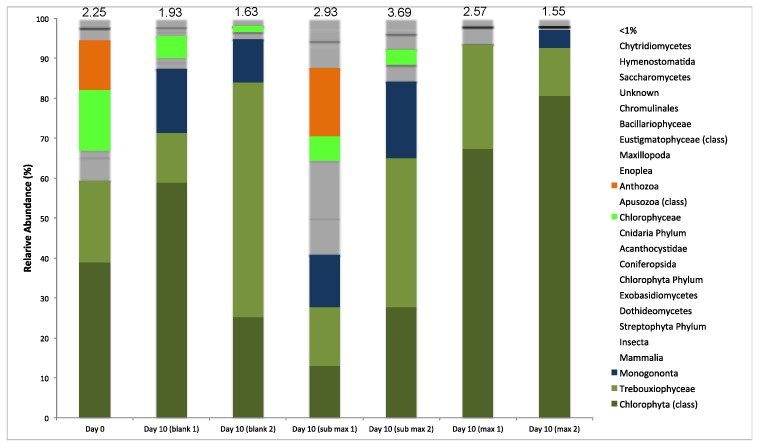
Relative gene abundance (%) of microorganisms identified within WRP sample, control (no harvesting), sub-maximum harvesting, and maximum harvesting (in biological replicates). The 16S rDNA bacterial abundance is shown at a genus level except when indicated (**top**) and 18S rDNA eukaryotic abundance is shown at a class level except when indicated (**bottom**). Algae (eukaryotic microalgae and prokaryotic cyanobacteria) are shown in shades of green. The Shannon diversity indices based on OTUs are shown at the top of each column.

In regards to the microbial community composition, Figure 4 shows the relative abundance of bacterial and eukaryotic biota in the WRP (day 0), where no harvesting (control) was performed, and where sub-maximum and maximum harvesting (after 10 days) were undertaken. The average number of reads for these samples was 67,687 with a mean length of 414 base pairs. A scatter plot of coordinates (PCoA) groups diversity between biological replicates, providing confidence in the results (Figure S2). Most notably, bacterial diversity was dominated by the cyanobacterium *Synechococcus* in the WRP (day 0) and the control samples (day 10). Sub-maximum harvesting reduced this by more than 2-fold, and their presence was all but eliminated in the maximum harvesting samples. This resulted in an increase in diversity, reflected by an increased Shannon diversity index. Interestingly, the population identities were very different between the replicate maximum harvesting samples after 10 days incubation (Figure 4). Eukaryotic diversity showed clear dominance from the Chlorophyta class in all samples (Figure 4). These results were less clear to interpret due to the high number of sequences that were not matched and the high level of variation between biological replicates illustrated by PCoA analysis (Figure S2). 

Algal blooms are viewed as problematic principally due to the production of toxins by certain species, mostly cyanobacterial. However, the impact of blooms on aquatic biodiversity is also a major cause for concern. The data presented here provides insight into how the microbial community (and function) changes from an undesirable alga-dominated system (pre-harvesting) to a bacterial/algae-based system (post-harvesting), particularly with maximum harvesting. There was no evidence that the parameters applied targeted specific microbial groups. The variability between replicates in the bacterial community 10 days after maximum harvesting demonstrates the complexity of microbial ecological succession in aquatic environments [52]. 

### 3.4. A Full Size Modular eFLOAT System

Although the resource analysis has detailed a number of useful products that could be harvested from the algal biomass, in most scenarios the overriding driver for biomass harvesting from the water body is for remediation i.e., the algal removal is a preventative or responsive ecosystem treatment. Given the scale of many of the components of the system, it is interesting to consider a modular eFLOAT pontoon-style system that would be capable of being flexibly scaled up or down, to meet the size requirements of a particular lake. Robust construction, ability to handle a wide range of weather conditions, low operational costs, and ease of repair, are all important design considerations for the modular units. 

**Figure 5:**
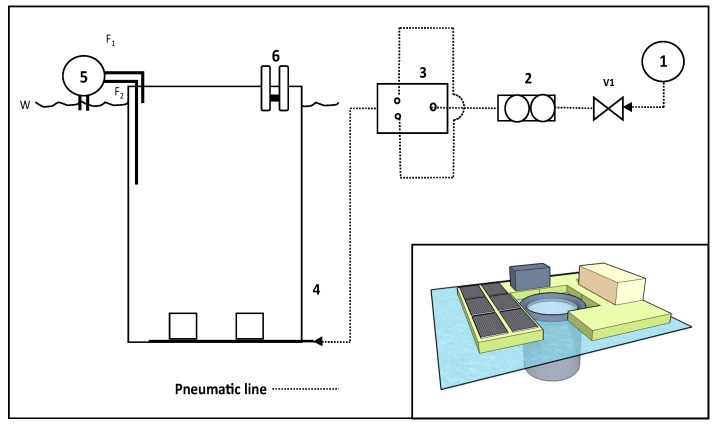
eFLOAT process based on a modular pontoon design (Inset: 3D eFLOAT design). Full capital expenditure (CAPEX) and operating expenditure (OPEX) figures are given in Table 2. 1. Compressor (50 L air compressor, 7.1 CFM, 1.5 HP, 1.1 kW, 110 V); 2. Rotameter; 3. Fluidic oscillator (Pressure drop of 150 mbar); 4. eFLOAT tank (24 m^3^ liquid capacity, 10% ullage for height, dimensions: 3 m diameter, 3.5 m height. 24 L·min^−1^ total air flow for the tank. 2 × 0.4 μm MBD600 diffusers. 12 L·min^−1^ average flow. 18 L·min^−1^ maximum rated flow; 5. Pump—Introducing and removing water from the eFLOAT tank, 320 W; 6. Skimmer—Recovering microalgal biomass, 27 kW; V_1_. Shutdown valve; F_1_ (Flow in); F_2_ (Flow out); W (Water level).

In terms of scale for a batch treatment enclosure for each pontoon, Zimmerman et al. provide a useful reference point, where a pilot plant tank of 24,000 L was used to investigate the efficacy of membrane-based microbubbles to increase gaseous mass transfer [17]. This provides a reasonably sized volume to treat in a batch mode with eFLOAT. Laboratory-based analysis suggests that eFLOAT air requirements is 1 mL·min^−1^ to treat 1 L, which, when scaled up to a treatment volume of 24,000 L, would equate to an air supply requirement of 24 L of air min^−1^ at a pressure of 3 bar(g) (Figure 5). The air volume and pressure to accommodate this airflow would be covered by a 7.1 cfm (201 L·min^−1^) compressor with a 110 V input and 1.1 kW load rating. The airflow rating at 3 bar(g) will be approximately 25% of this value, which equates to 50 L·min^−1^. An additional electrical load would be the pond skimmer that floats within the enclosure and skims the floating algae to a separation tank at the appropriate time (depending on bloom size) during each batch process, as well as a pump to deliver and remove water from the eFLOAT tank. The overall timing of the harvesting process for 24,000 L is predicted to be 30 min, with an additional 48 min to introduce and also to remove the water from the eFLOAT tank (total = 126 min). The addition of flocculant during eFLOAT would be an extra consideration, and we have identified 5 mg·L^−1^ chitosan as sufficient with the WRP sample within the laboratory scale rig. The cost of chitosan is estimated at £30 Kg^−1^ although this varies by quality and quantity purchased. Full capital expenditure (CAPEX) and operating expenditure (OPEX) figures are provided in Table 2 and for treatment of the WRP (assuming 7500 m^3^ water) for the full size modular eFLOAT system. A treatment of this volume would cost less than £2000 over a week. Moreover, costs can be reduced further using locally sourced flocculants, for example, modified local sands [53]. The exact efficiency of the process over a season, and therefore total operating costs, would vary depending on the type of bloom (i.e., species present, concentration of cells, etc.), chemistry of the water, and environmental conditions.

These modular eFLOAT pontoons would ideally be powered by renewable energy, e.g., solar PV panels, and be deployed and managed by a single operator to harvest the biomass. In this manner, these could be quickly scaled and deployed to areas where algal blooms are prevalent. This design could be accommodated by 6–8 solar panels of 250+ W each, depending on the trade-off between capital costs and working timeframe in a given day. This is also dependent on the insolation values wherever in the world the system is deployed. The physical size of the solar panels would be somewhere between 8 m^2^ and 12 m^2^, dependent on the type of solar panel, and this is shown in the inset graphic of a modular pontoon in Figure 5. 

Although most of the modular components of the eFLOAT pontoon concept are commercially available, the overall system has not been verified or optimized. Further research is required to establish how the airflow is impacted by changing the tank dimensions and by optimizing the system components and operating protocols for a range of different input conditions. Interesting research questions arise around the least number of solar panels that could be used, and the trade-off between the number of solar panels, pump size, air tank volume, and the rate at which a batch process could be undertaken.

## 4. Conclusions

This laboratory-scale study demonstrates that a combination of chitosan-induced flocculation and eFLOAT has the potential to remove polluting algal biomass from natural water systems, and a modular pontoon has been theorized. The application would be particularly useful in open lake or pond systems, similar to the WRP, where PO_4_^3−^ concentrations are very high (mg·L^−1^ range). Therefore, this application could provide a treatment methodology where polluting PO_4_^3−^ is removed within algal biomass. We argue that the combined use of an environmentally-friendly flocculant, together with low energy requirements, would allow a scaling up the of the eFLOAT technology with a pontoon-style harvesting system. Although recovering a lake to healthy status would out-weigh the economic benefits of recovering resources, these do provide an additional incentive, and the technology could be applied to target specific microbial biomass suitable for valorization.

## Figures and Tables

**Table 1:** Detailed physicochemical characterization of water retention pond (WRP) during a bloom event (19th May 2014).

Parameter	Value	SD	Unit
Temperature	18.1	-	˚C
pH	7.01	-	
DO	9.86	-	mg·L^−1^
Conductivity	302.1	-	µS·cm^−1^
Salinity	0.17	-	PSU
Dry Weight	0.13	0.003	g·L^−1^
Chlorophyll *a*	443.23	-	µg·L^−1^
F^−^	0.00	0.00	mg·L^−1^
Cl^−^	26.49	0.21	mg·L^−1^
NO_2_^−^	9.72	0.02	mg·L^−1^
SO_4_^2−^	6.00	0.04	mg·L^−1^
Br^−^	0.00	0.00	mg·L^−1^
NO_3_^−^	2.59	0.04	mg·L^−1^
PO_4_^3−^	6.11	0.02	mg·L^−1^
Na^+^	16.85	0.10	mg·L^−1^
NH_4_^+^	0.48	0.02	mg·L^−1^
K^+^	4.02	0.06	mg·L^−1^
Mg^2+^	9.15	0.00	mg·L^−1^
Ca^2+^	37.42	0.01	mg·L^−1^

**Table 2:** CAPEX and OPEX of eFLOAT system based on one modular system treating 24,000 L water in 30 min. (Figures are given as a guideline only).

Item	CAPEX (£)	OPEX (£)	Application Time (min)	Full Cost for WRP (7.5 ML)
Compressor	200	0.83	30	259
Rotameter	60	0	30	0
Fluidic oscillator	100	0	30	0
eFLOAT tank	1420 *	0	n/a	0
Pumps	1000	0.077	96	24
Skimmer	1000	1.20 **	30	375
Chitosan	n/a	30 Kg^−1^	5	1125

* 2 diffusers (£210 each) + tank (£1000); ** 16 kW for skimmer pump; Assuming 15p kWh^−1^.

## Supplementary Materials

The following are available online at www.mdpi.com/2079-7737/7/1/4/s1. Table S1: Complete composition of artificial freshwater growth medium. Table S2: A summary of FAME profiles between different microalgae species mostly focussing on recent studies. The studies were selected based on similar methodologies applied to sample processing steps, catalysts, GC columns and GC conditions. NB. Percentages will vary depending on the number of FAMEs quantified in each experiment and only the major FAMEs detected are shown for clarity in the comparison (see citation for more details of exact experimental procedures). Table S3: Chlorophyll *a* measurements during eFLOAT long-term experiment. Figure S1: 1 litre eFLOAT Rig using during laboratory tests. Figure S2: Scatter plot of coordinates (PCoA) to show relative abundance (%) variation between identified genus/class in biological replicate samples (A) Bacterial (16S rDNA sequencing) diversity (B) Eukaryotic (18S rDNA sequencing) diversity. HM: half max harvesting, M: max harvesting. Figure S3: Rarefaction plots for bacterial primers (28F-519R) and eukaryotic primers (565-981).
