# Supplementary material for: Harvesting Environmental Microalgal Blooms for Remediation and Resource Recovery: A Laboratory Scale Investigation with Economic and Microbial Community Impact Assessment
Source: Biology (Basel). 2017 Dec 29;7(1):4. doi: 10.3390/biology7010004 (PMC5872030; doi:10.3390/biology7010004)
Supplement: Supplementary file 1 [file biology-07-00004-s001.pdf]

Supplementary Materials:

Harvesting Environmental Microalgal Blooms for Remediation and Resource Recovery: A Laboratory Scale Investigation with Economic and Microbial Community Impact Assessment

Jagroop Pandhal, Wai L. Choon, Rahul V. Kapoore, David A. Russo, James Hanotu, I. A. Grant Wilson, Pratik Desai, Malcolm Bailey, William J. Zimmerman and Andrew S. Ferguson

Table S1: Complete composition of artificial freshwater growth medium.

Component	Concentration (mg/L)
$NaHCO_3$	192
$MnCl_2.4H_2O$	0.18
$MgSO_47H_2O$	115
KCl	0.45
H_2SeO_3	0.0016
$Ca(NO_3)_24H_2O$	0.8
NH_4Cl	1
KH_2PO_4	0.025
K_2PO_4	0.025
$ZnSO_4.7H_2O$	0.022
$Na_2EDTA.2H_2O$	0.5
H_3BO_3	0.114
$FeSO4.7H_2O$	0.05
$CuSO_4.5H_2O$	0.016
$CoCl_2.6H_2O$	0.016
$(NH_4)_6Mo7O_{24}.4H_2O$	0.011

Table S2: A summary of FAME profiles between different microalgae species mostly focussing on recent studies. The studies were selected based on similar methodologies applied to sample processing steps, catalysts, GC columns and GC conditions. NB. Percentages will vary depending on the number of FAMEs quantified in each experiment and only the major FAMEs detected are shown for clarity in the comparison (see citation for more details of exact experimental procedures).

**Species**	**Author**	**Year**	**C14:0**	**C16:0**	**C16:1**	**C16:2**	**C16:3**	**C17:0**	**C18:0**	**C18:1**	**C18:2**	**C18:3**	**C18:4**	**C20:4**	**C20:5**	**C22:6**	**Others**
**Published results**																	
Chlamydomonas reinhardtii	Talebi at al.	2013	-	23.8	1.9	-	-		4.4	19.7	6.6	25.9	-	-	-	-	1.2
Chlorella pyrenoidosa	Montes et al.	2011	0.7	17.3	0.8	7.0	9.3		1.2	3.3	18.5	41.8	-	-	-	-	-
Chlorella sp. 227	Sunja et al.	2010	-	22.8	-	-	-		-	8.3	17.2	52.0	-	-	-	-	-
Chlorella vulgaris	Laurens et al.	2012	0.2	18.3	12.3	6.1	0.3		1.2	18.9	15.1	24.0	-	0.0	0.0	-	0.9
Dunaliella primolecta	Lang et al.	2011	0.6	26.0	0.9	-	-		1.6	16.3	7.0	38.7	0.6	-	-	-	-
Dunaliella tertiolecta	Chen et al.	2011	-	28.1	0.0	2.8	1.4		0.6	19.3	14.7	33.2	-	-	-	-	-
Emiliana huxleyi	Lang at al.	2011	18.8	10.3	-	-	-		10.8	42.2	-	-	8.7	-	-	9.2	
Heterosigma akashiwo	Lang et al.	2011	6.6	40.0	12.7	4.0					4.5	6.7	5.2	3.5	14.8	-	-
Isochrysis galbana	Fidalgo at al.	1998	14.4	12.6	19.4	0.5	0.2		0.4	3.1	0.7	1.6	15.2	-	22.2	7.9	0.3
Nannochloris sp.	Lang et al.	2011	13.3	17.8	-	-	-			23.9	10.8	28.2	6.1	-	-	-	-
Nannochloropsis	Koberg at al.	2011	6.6	42.8	27.3	-	-	0.4	1.0	9.1	1.3	0.4	-	-	4.9	-	3.0
Nannochloropsis oculata	Converti at al.	2009	-	62.0	-	-	-		11.0	5.0	8.0	15.0	-	-	-	-	-
Nannochloropsis	Wagenen	2012	7.0	19.0	30.0	-	-		-	8.0	-	-	-	6.0	26.0	-	-

Salina	at al.																
Parietochloris incisa	Lang at al.	2011	0.0	19.8	-	5.2			18.2	10.2	14.3	14.3	-	14.0	4.3	-	-
Pavlova lutheri	Lang at al.	2011	10.1	11.1	26.3	-	-			5.2	0.6	0.5	9.1	0.3	18.0	9.7	-
Phaeodactylum tricornutum	Lang et al.	2011	6.7	14.7	43.6	2.0	-		-	15.8	0.5	0.4	1.1	-	14.4	0.7	-
Scenedesmus obliquus	Breuer et al.	2013	2.0	27.0	45.0	-	1.0		2.0	3.0	1.0	<1	<1	1.0	14.0	-	2.0
Scenedesmus sp.	Talebi et al.	2013	-	15.6	4.1	-	-		3.0	15.2	7.0	23.0	-	-	-	-	7.5
Schizochytrium limacinum	Johnson at al.	2009	5.3	56.5	-	-	-		-	-	-	-	-	-	-	29.7	5.2
Thalassiosira weissflogii	Borges et al.	2011	6.8	17.2	24.3	-	18.1		2.5	10.0	-	-	-	-	17.3	1.6	3.7
Thalassiosira weissflogii	Lang et al.	2011	8.8	36.6	40.5	-	-		-	14.0		-	-	-	-	-	-
Wild algae	Krohn et al.	2011	-	47.1	-	-	-		7.0	-	-	-	-	-	-	-	-
**This study**																	
NBAS	Pandhal et al.	2015	9.8	28.1	1.6	-	-	2.9	5.0	8.9	12.2	31.4	-	-	-	-	-
SBAS	Pandhal et al.	2015	7.2	53.7	4.4	-	-	2.5		4.0	21.5	0.2	-	-	-	0.3	6.1
ESTH	Pandhal et al.	2015	8.2	30.3	0.6	-	-	0.9	9.2	2.9	7.6	16.4	-	-	-	4.0	19.8
NLT	Pandhal et al.	2015	0.5	52.9	0.9	-	-	0.3	3.0	3.6	12.5	23.8	-	-	-	-	2.5
WRP	Pandhal et al.	2015	19.3	18.0	1.6	-	-	0.7	13.7	8.7	4.3	22.1	-	-	-	0.3	11.1

Table S3: Chlorophyll a measurements during eFLOAT long-term experiment.

Sample	Time post harvest (days)	Chl a (μg L^{-1})	Average Chl *a* (μg L^{-1})	Variation (μg L^{-1})	Decrease %	Average %	Variation %
WRP	0	443.23					
Blank 1	10	404.37			8.8		
Blank 2	10	387.12	395.7	12.2	12.7	10.7	2.8
Sub-max 1	10	145.37			67.2		
Sub-max 2	10	162.91	154.1	12.4	63.2	65.2	2.8
Max 1	10	95.82			78.4		
Max 2	10	62.1	79.0	23.8	86.0	82.2	5.4

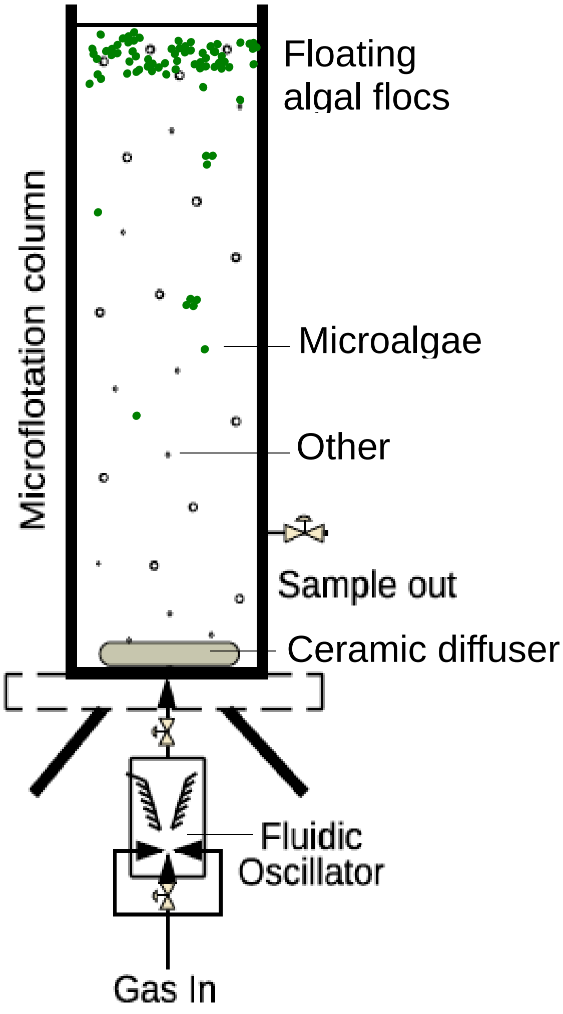

Figure S1: 1 litre eFLOAT Rig using during laboratory tests

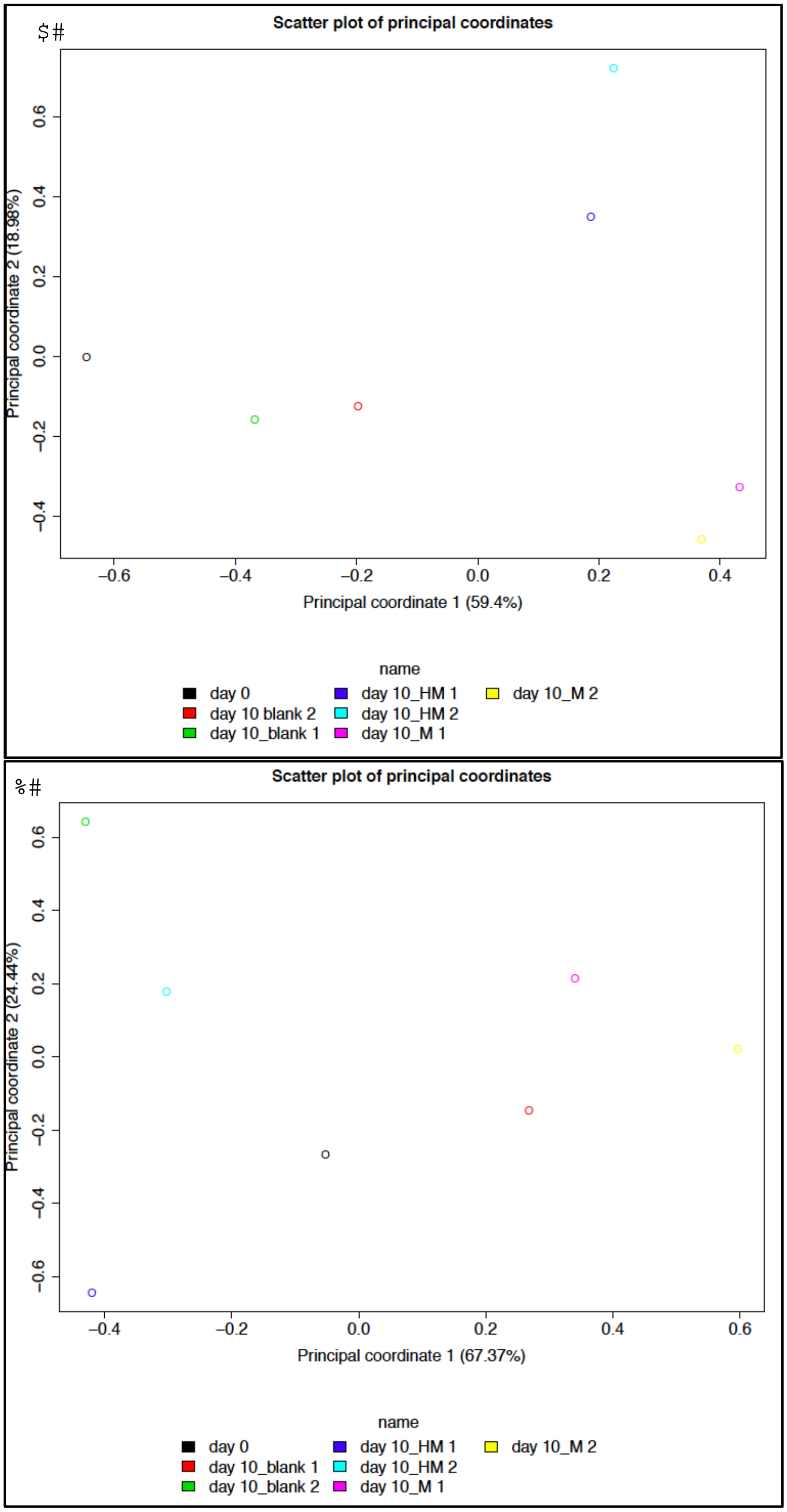

Figure S2: Scatter plot of coordinates (PCoA) to show relative abundance (%) variation between identified genus/class in biological replicate samples (A) Bacterial (16S rDNA sequencing) diversity (B) Eukaryotic (18S rDNA sequencing) diversity. HM: half max harvesting, M: max harvesting.

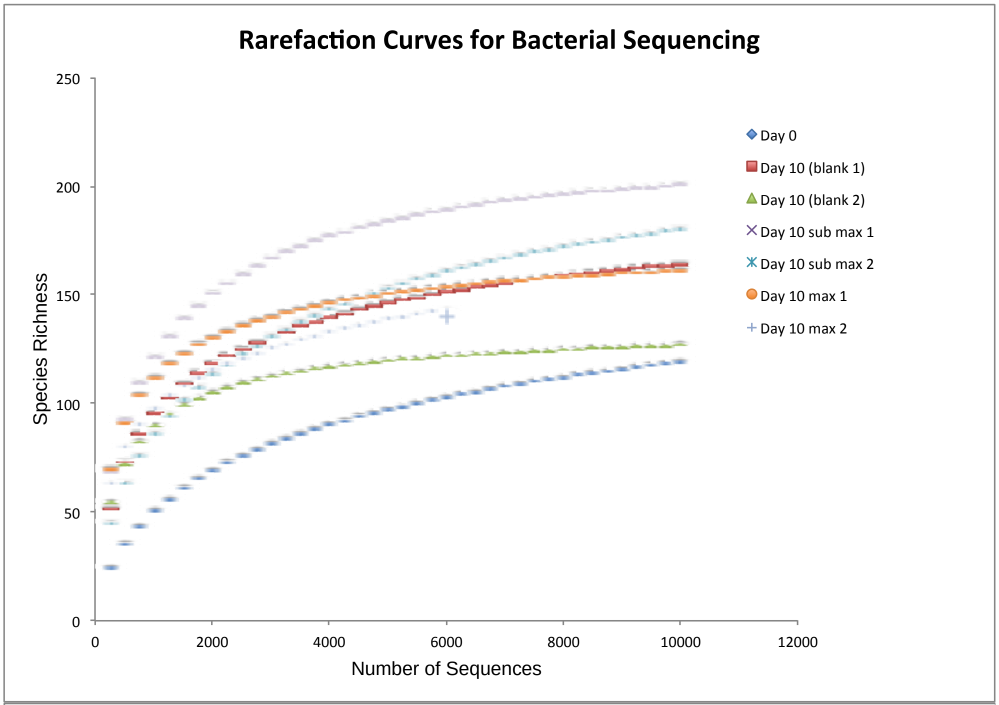

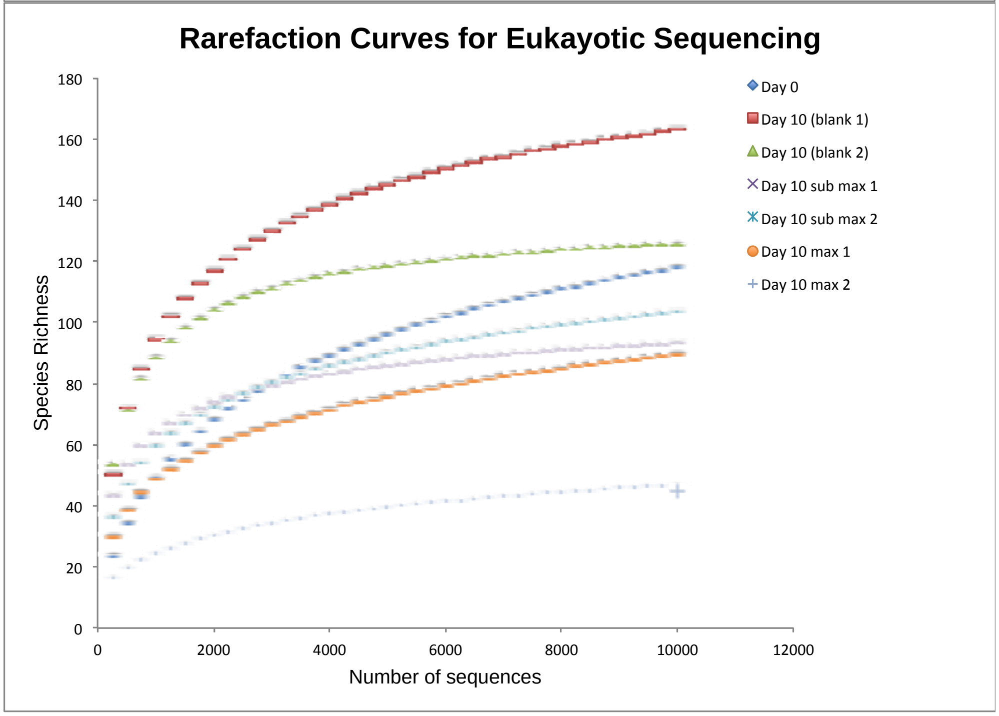

Figure S3: Rarefaction plots for bacterial primers (28F-519R) and eukaryotic primers (565-981).
